# Supplementary material for: Implicit preference for human trustworthy faces in macaque monkeys
Source: Nat Commun. 2018 Oct 30;9:4529. doi: 10.1038/s41467-018-06987-4 (PMC6207650; doi:10.1038/s41467-018-06987-4)
Supplement: Supplementary file 1 — Costa et al Supplementary Information [file 41467_2018_6987_MOESM1_ESM.pdf]

Implicit preference for human trustworthy faces in macaque monkeys

‘Costa et al.’

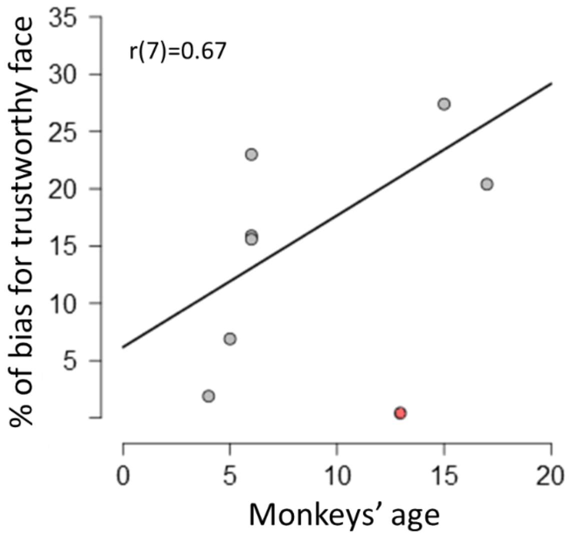

Supplementary Fig. 1. Correlation between monkeys' age and percentage of trust bias**.** Percentage of bias for trustworthy-associated facial cues ($r(7)=-0.67$, $p<0.05$) positively correlated with the age of the monkey, i.e., the older the monkey the larger the bias toward trustworthy–associated facial cues. Each point corresponds to a monkey. For this analysis the only female monkey outlier of the group was excluded though we left her position on the plot (in red) for illustration purposes.

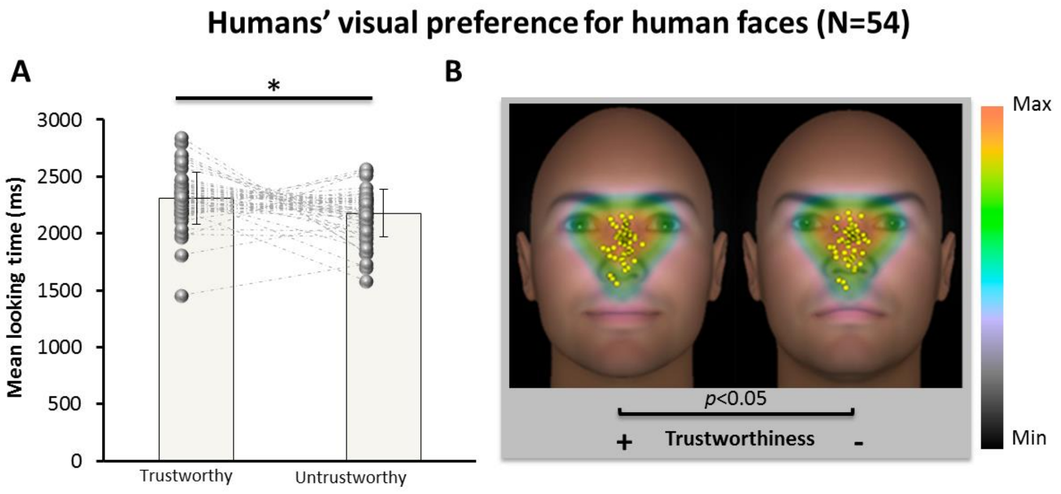

Supplementary Fig. 2: Human's looking preference for trustworthy vs. untrustworthy faces. Plot **(A)** shows significantly longer mean looking times at trustworthy than untrustworthy. Error bars indicate S.E.M. across 54 subjects. *$p<0.05$, paired-sample *t*-test. The average barycenter of fixation was located in the region surrounding the eye region and the nose **(B)**. Faces are selected from the Todorov's Database of trustworthy faces [4-5].

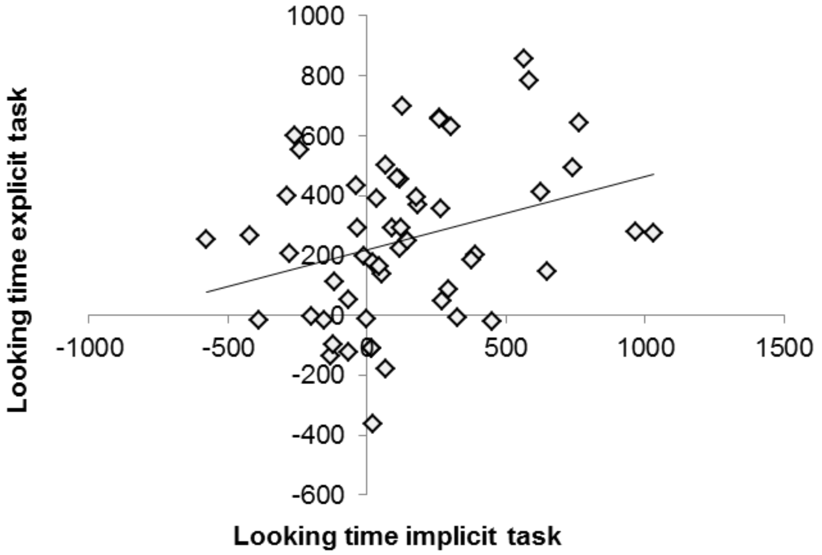

Supplementary Fig. 3 Implicit and explicit looking time for trust. We observed a positive correlation (r=0.30; p=0.027, Pearson correlation) between difference in looking time (trustworthy - untrustworthy) during the implicit and the explicit task. Subjects that looked more the trustworthy faces during the implicit task also looked more the same faces when they had to explicitly select the trustworthy face. This result supports that eye movements in humans predict their explicit judgments.

Supplementary Note 1.

Pupil size in humans for trustworthy and untrustworthy faces. Pupil size data (n=20). was not recorded in monkey subjects. In human, to assess whether a difference in pupil dilation existed between the two conditions (trustworthy –untrustworthy) we analyzed pupil data in our participants. First, extracted pupil data (pupil size in pixels) was linearly interpolated to fill missing values from blinks. Then, pupil data were band-pass filtered (between 0.175Hz and 3.5Hz) for each participant separately, on his/her entire time course to remove the slow derivation of pupil size and some transient artifacts that may appear in measurements [1,2].

During the task, participants' mean pupil size measured before filtering was mean±s.d = 3.7± 0.67 mm. Then, for each participant, the filtered pupil size values were extracted whenever the gaze was in the trustworthy-face region of interest and averaged across trials. The same procedure was used to calculate the magnitude of pupil size modification over untrustworthy faces. First, we tested whether pupil size was different from the average pupil size when gaze was over the face area and second, if pupil size was different over the two regions of interest (trustworthy vs untrustworthy). Results indicate that humans constricted their pupil both when they gazed at trustworthy and untrustworthy faces (trustworthy: -0.041 ± 0.026, $t(19) = -6.72$, $p < 0.001$; untrustworthy: -0.038 ± 0.02, $t(19) = -7.16$, $p < 0.001$), an effect most likely driven by attention[1,2] although a brightness difference between the face stimuli and the dark background of the visual display cannot be excluded. No significant differences were found between the two faces category (paired sample *t*-test; $t(19) = 0.087$, $p = 0.196$).

Supplementary Note 2.

Spontaneous perception and judgment of trust in humans with 5000ms stimuli exposure. A preliminary experiment was performed with fifty-four healthy subjects, (27 women, 26.9 ± 5.9 years) using a 5 seconds exploration duration similar to the study of Méary and colleagues (2014) but with an otherwise identical task design as in the main implicit visual preference experiment.

Subjects showed a significant bias in favor of the trustworthy (2311.96 ± 228.72 ms) face category compared to the untrustworthy (2177.11 ± 208.75 ms, paired sample t-test; $t(53) = 2.96$, $p< 0.005$, $\eta^2 = 0.14$) (Supplementary Fig. 2). They also exhibited a significantly higher number of fixations in favor of the trustworthy faces (6.66 ± 1.60; untrustworthy: 6.33 ± 1.48; paired sample *t*-test; $t(53)=2.44$; CI 0.058 - 0.59; $p = 0.0179$).

In order to determine whether longer looking times were actually related to the perception of trustworthiness, in a subsequent separate session, the same 54 human subjects were explicitly asked to select the most trustworthy face while their eye movements were again recorded. We found that difference in mean looking times between trustworthy/untrustworthy was significantly correlated between the first (implicit) and the second (explicit) condition ($r = 0.30$, $p < 0.05$, Pearson correlation) (Supplementary Fig. 3). This shows that humans' spontaneous looking times toward trustworthy faces in the non-instructed viewing task is a reliable implicit marker of trustworthiness detection.

Supplementary Note 3.

Dominance experiment. To demonstrate monkeys' selective preference for trustworthy faces we designed a new experiment where we tested the animals' perception of dominant/submissive faces. As it was the case in the trust experiment we presented dominant and subdominant faces taken from Todorov's database. The stimuli used were 50 computer-generated male faces created using the software FaceGen Modeller (http://facegen.com, version 3.1). Each facial identity varied along the dimension of dominance on both shape and reflectance. The Dominance database is composed of facial identities manipulated to create seven versions (-3 SD, -2 SD, -1 SD, 0 SD, 1 SD, 2 SD, and 3 SD) on dominance[4-5]. For the current study, we selected from the 25 identities the two most extreme versions (-3 SD and +3 SD), resulting in 25 pairs. The same monkeys (N=8) performed the preferential looking paradigm. Monkeys spent 574,51ms (SD=193,88) looking at submissive human faces and 447,01 ms (SD=209,33) looking at the dominant ones, but the difference between the two

types of face was not significant (paired sample t-test; $t(7) = 1.66$; $P = 0.13$). This result suggests that monkeys' do not robustly distinguish between dominant and subdominant human faces.

Supplementary References:

1. Cohen, N., Moyal, N. & Henik, A. Executive control suppresses pupillary responses to aversive stimuli. Biol. Psychol. 112, 1–11 (2015).

2. Hoehl, S., Hellmer, K., Johansson, M. & Gredebäck, G. Itsy bitsy spider…: infants react with increased arousal to spiders and snakes. Front. Psychol. 8, 1710 (2017).

3. Méary, D., Li, Z., Li, W., Guo, K. & Pascalis, O. Seeing two faces together: preference formation in humans and rhesus macaques. *Anim. Cogn.* **17,** 1107–1119 (2014).

4. Todorov, A., Dotsch, R., Porter, J. M., Oosterhof, N. N. & Falvello, V. B. Validation of Data-Driven Computational Models of Social Perception of Faces. *Emotion* **13,** 724–738 (2013).

5. Todorov, A. & Oosterhof, N. Modeling Social Perception of Faces. *IEEE Signal Process. Mag.* **28,** 117–122 (2011).
